# The muscle twitch profile assessed with motor unit magnetic resonance imaging

**DOI:** 10.1002/nbm.4466

**Published:** 2021-01-06

**Authors:** Linda Heskamp, Matthew G. Birkbeck, Roger G. Whittaker, Ian S. Schofield, Andrew M. Blamire

**Affiliations:** ^1^ Newcastle University Translational and Clinical Research Institute (NUTCRI) Newcastle University Newcastle upon Tyne UK; ^2^ Newcastle Biomedical Research Centre Newcastle University Newcastle upon Tyne UK; ^3^ Northern Medical Physics and Clinical Engineering Freeman Hospital, Newcastle upon Tyne NHS Foundation Trust Newcastle upon Tyne UK

**Keywords:** contraction time, diffusion weighted imaging, Motor unit, Motor unit MRI, muscle twitch, phase contrast imaging

## Abstract

Localised signal voids in diffusion‐weighted (DW) images of skeletal muscle have been postulated to occur as a result of muscle fibre contraction and relaxation. We investigated the contrast mechanism of these signal voids using a combination of modelling and experimental measurements by employing DW and phase contrast (PC) imaging sequences. The DW signal and PC signal were simulated for each time point of a theoretical muscle twitch. The model incorporated compaction (simulating actively contracting muscle fibres) and translation (simulating passively moving surrounding fibres). The model suggested that the DW signal depended on contraction time and compaction whereas the PC signal depended on contraction time, compaction and translation. In a retrospective study, we tested this model with subgroup analyses on 10 healthy participants. Electrical nerve stimulation was used to generate muscle twitches in lower leg muscles; the resulting force was measured using an MR‐compatible force transducer. At current levels causing a visible muscle twitch (~13 mA), the width of the first signal drop in the DW signal (mean ± SD: 103 ± 20 ms) was comparable with the force contraction time (93 ± 34 ms; intraclass correlation coefficient [ICC] = 0.717, *P* = .010). At current levels activating single motor units (~9 mA), the contraction time determined from the DW signal was 75 ± 13 ms and comparable with the PC contraction time (81 ± 15 ms; ICC = 0.925, *P* = .001). The maximum positive velocity was 0.55 ± 0.26 cm/s and the displacement was 0.20 ± 0.10 mm. Voxel‐wise analysis revealed localised DW changes occurring together with more widespread phase changes. In conclusion, local signal attenuations in DW images following muscle fibre activation are primarily caused by compaction. The PC sequence also detects translating muscle tissue being passively pulled. The magnitude of the changes in DW and PC images depends on the twitch's contractile properties and percentage contraction. DW imaging and PC imaging can therefore measure twitch profiles of skeletal muscle fibres.

## Graphical abstract

Muscle fibre contraction induces signal changes in diffusion weighted and phase contrast images, which we used to extract the twitch time profile of individual motor units with a technique called motor unit MRI (MUMRI). Using modelling and experimental data, we showed that these signal changes depend on the contractile characteristics of the muscle twitch. Furthermore, we found that the diffusion weighted signal represents the active contraction, and the phase contrast measured velocity also detects passively moving muscle fibres.

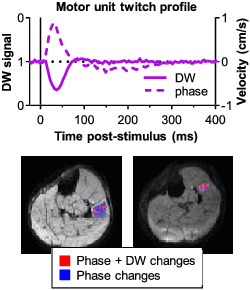

**Abbreviations used**
ALS: amyotrophic lateral sclerosis
Bipolar PC: bipolar phase contrast gradient
DW: diffusion‐weighted
EDL: extensor digitorum longus
ICC: intraclass correlation coefficient
I_muscle_: electrical stimulation current producing a visible muscle twitch and detectable force
I_singleMU_: electrical stimulation current that activated single motor units
MUMRI: motor unit magnetic resonance imaging
PC: phase contrast
PGSE‐DWI: pulsed gradient spin echo diffusion‐weighted imaging
PL: peroneus longus
RF: radiofrequency
ROI: region of interest
SOL: soleus
TA: tibialis anterior
VENC: velocity‐encoding

## 1 INTRODUCTION

Each muscle has many motor units that interdigitate across the muscle and allow gross and fine control of muscle function.^1^, ^2^, ^3^ A motor unit comprises a lower motor neuron, with a cell body situated in the spinal cord with an axon extending into the muscle, and the skeletal muscle fibres that this motor neuron innervates.^2^, ^3^, ^4^ Changes in motor unit structure or performance with ageing or disease can have a significant impact on muscle control. For example, amyotrophic lateral sclerosis (ALS) is a motor neuron disease and is hallmarked by progressive loss of motor units causing changes in the motor unit function and morphology. This is followed by histopathological muscle changes like fluid shifts, fat infiltration and atrophy, leading to muscle weakness.^1^

Standard MR imaging techniques such as T1‐weighted imaging, DIXON methods and Short Tau Inversion Recovery (STIR) allow for imaging of muscle degeneration, fat infiltration and atrophy. These techniques inform us about the irreversible histological consequences of motor neuron degeneration but tell us nothing about the preceding functional changes to the motor units. Spontaneous, short‐lived signal voids have been observed in diffusion‐weighted (DW) images of skeletal muscle.^5^, ^6^, ^7^, ^8^, ^9^ It has been postulated that this attenuation of the MR signal arises as a result of intravoxel reordering of tissue water due to contraction of muscle fibres in a motor unit rather than simple diffusion.^5^, ^6^, ^8^, ^9^ The muscle DW scans have been developed into an imaging approach to assess motor unit function and morphology; we call this technique motor unit MRI (MUMRI). MUMRI has been applied in patients with ALS to detect fasciculation, a process of pathological spontaneous firing of individual motor units. In that study, the signal voids occurred more frequently in ALS patients compared with healthy volunteers.^5^ MUMRI has also been applied following electrical stimulation of the common peroneal nerve and posterior tibial nerve to image single motor unit morphology in healthy volunteers.^10^

If the timing of the electrical nerve stimulation is systemically shifted in relation to the imaging acquisition window a signal curve can be extracted with the shape and time course that seems to closely match the twitch‐tension measurements in electrically stimulated motor units.^5^ This suggests that MUMRI can be used to measure the contractile properties of single motor units; however, this has yet to be validated. To do so, application of a phase contrast (PC) sequence becomes of interest as this allows quantification of the contractile velocity and displacement of the muscle fibres.^11^, ^12^

The aforementioned observations strongly indicate that the observed signal attenuation on DW MUMRI images are caused by reordering of water molecules by muscle fibre contraction and relaxation. However, it is still unclear how the mechanism of contraction and the muscle fibres' contractile properties influence the measured attenuation in the DW signal. Therefore, the first aim of this study was to investigate how different muscle twitch profiles affect both the DW signal and PC signal using a computational model and by comparing this model with experimentally measured images. Second, we aimed to measure the twitch profile and assess the contractile properties of single motor units using both DW and PC sequences.

## 2 MATERIALS AND METHODS

### 2.1 Theoretical model

#### 2.1.1 Model description

**FIGURE 1:**
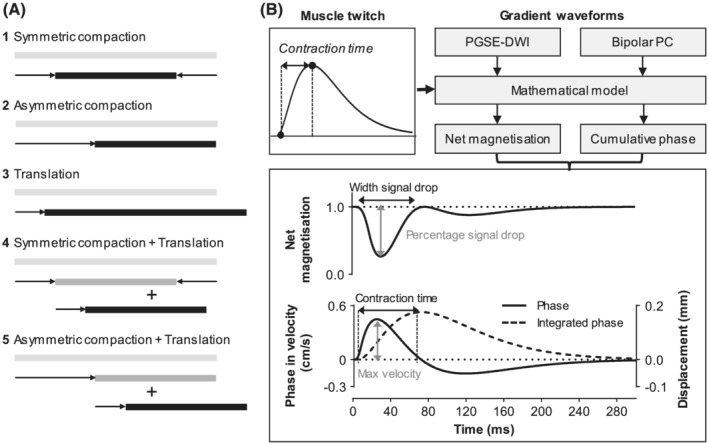
Computational model. A, Five theoretical situations of muscle contraction. The light grey bar represents the muscle fibre (or set of elements of magnetisation) before contraction and the black bar the final result after contraction. B, Schematic overview of the computational model, including the model input and output (top), and the calculated outcome measures from the simulated net magnetisation and cumulative phase (bottom). PGSE‐DWI, pulsed gradient spin echo diffusion‐weighted imaging; Bipolar PC, bipolar phase contrast gradient

A computational simulation was written in Matlab 2019a (Mathworks, Natick, MA, USA) to study how muscle fibre contraction affects the signal in MUMRI images. In this simple model we considered a 1D set of 10 001 points equally spaced across the thickness of the MR imaging slice, assuming the slice is perpendicular to the muscle fibres and that the set of points lie along the muscle fibre. Each point represents an element of magnetisation, from which the effect of movement and pulse sequence parameters could be studied. These points were then subjected to a model of muscle contraction. Skeletal muscles comprise multiple overlapping motor units, with actively contracting muscle fibres interdigitating with and surrounded by physically coupled neighbouring, inactive fibres. We modelled active contraction as a compaction of the set of points (ie, the set of points contracts and the points move closer to each other). Depending on the position of the fibre in relation to the imaging slice, this could be symmetrical or asymmetrical. We modelled passive movement of adjacent inactive fibres as translation (ie, all points moved an equal amount and the distance between the points remained the same). Regions of overlapping active and inactive fibres were modelled by a combination of the two. This gives five simplified situations of contraction (Figure 1A):

1. Contraction with symmetric compaction.
2. Contraction with asymmetric compaction.
3. Contraction with translation.
4. Contraction with symmetric compaction plus translation (situation 1 + 3).
5. Contraction with asymmetric compaction plus translation (situation 2 + 3).

Each model was then applied in the presence of two simulated gradient waveforms, pulsed gradient spin echo DWI Imaging (PGSE‐DWI) gradient waveform and Bipolar PC gradient waveform. The PGSE‐DWI gradient waveform was simulated for a b value of 20 s/mm^2^ with a diffusion time (Δ) of 16.9 ms and gradient duration of δ = 2.2 ms (equivalent to a velocity‐encoding [VENC] sensitivity of 0.14 cm/s), based on typical waveforms from our previous work and the experimental work described later.^5^, ^10^ The Bipolar PC gradient waveform was simulated for a VENC sensitivity of 1.3 cm/s (equivalent to a diffusion b value of 5 s/mm^2^ with Δ = 9.13 ms and δ = 4.57 ms).

The simulation was supplied with three basic elements: a theoretical muscle twitch waveform, the simulated gradient waveform and the model of contraction (Figure 1B). The gradient waveform was stepped in time across the twitch waveform in steps of 0.1 ms (*t*_*s*_); this simulated the effect of moving the timing of a nerve stimulus relative to the encoding gradients of the sequence. The effect of the contraction on the spatial location of the magnetisation was calculated for each point in time using Equation 1:

(1) $$x_{\mathrm{po}s_{\mathrm{new}}}=\left[x_{\mathrm{po}s_{\text{orig}}}*\left(1-\left(w\left(t\right)*\alpha \right)\right)\right]-\left(w\left(t\right)*d*\beta \right),$$

where *x*_*pos_orig*_ is the original position of the point before the model of contraction was applied, *w*(*t*) is the temporal position on the twitch waveform (in ms) as a function of time, *α* is the percentage of compaction relative to the slice thickness, *d* is the slice thickness set in cm (set as 0.75 cm), and *β* is the percentage translation relative to the slice thickness. In case of translation, we assumed there was no loss of magnetisation by points moving outside the slice.

At the start of each simulation, each element of magnetisation was set to be entirely in the transverse plane and in phase. The instantaneous change in the phase of the magnetisation caused by the change in position during the contraction was then calculated using Equation 2:

(2) $${∆}_{\mathrm{ph}}=G\left(t\right)*x_{\mathrm{po}s_{\mathrm{new}}}*t_{s},$$

where *G*(*t*) is the gradient strength (in Hz/cm) at each time point across the gradient waveform and *t*_*s*_ is the sample time of 0.1 x 10^−3^ seconds.

Thereafter, the magnitude of the net magnetisation and its cumulative phase (*φ*_*ph*_) were calculated via the integrated sum of *M*_*x*_ and *M*_*y*_ at each location across the slice, given by Equations 3 to 6:

(3) $$M_{x}=\sum \cos\;2\pi \;{∆}_{\mathrm{ph}}$$

(4) $$M_{y}=\sum \sin\;2\pi \;{∆}_{\mathrm{ph}}$$

(5) $$M_{\mathrm{net}}=\frac{\sqrt{{M_{x}}^{2}+{M_{y}}^{2}}}{n}$$

(6) $$\varphi_{\mathrm{ph}}=\int \tan^{-1}\frac{M_{x}}{-M_{y}},$$

where *n* is the number of spatial points spaced across the imaging slice.

In this study, PGSE‐DWI was used to study the effect of the contraction on the net magnetisation, as would be the case for the DW MUMRI images (Figure 1B). The Bipolar PC was applied to assess the effect of the contraction on the cumulative phase, representing the phase images of a PC sequence. The cumulative phase signal was expressed in velocity using the definition that one phase cycle is equivalent to a velocity of 1.3 cm/s.

#### 2.1.2 Simulations

For the first simulations, net magnetisation and cumulative phase were simulated for a theoretical twitch profile with contraction times of 20, 30, 40, 60, 80, 100, 120, 140, 160, 180 and 200 ms, with 5% asymmetric compaction and no translation. The resulting cumulative phase signal, representing the velocity, was integrated to reconstruct the displacement of the theoretical twitch (Figure 1B). For each twitch, the width of the first drop in net magnetisation (in ms) and its magnitude (in %) were determined. Furthermore, for the cumulative phase, we determined the maximum positive velocity (in cm/s) and for the integrated cumulative phase we determined the twitch contraction time (in ms).

Thereafter, the percentage compaction *α* and percentage translation *β* were varied from 0% to 12% in steps of 0.5%, for symmetrical and asymmetrical compaction. A theoretical twitch with an 80‐ms contraction time was used, as this was most physiologically relevant.^13^ The percentage drop in net magnetisation and the maximum positive velocity were determined and related to the percentage compaction and translation.

### 2.2 Participants

For the experimental part of this study, we performed retrospective subgroup analyses on the data of 10 healthy participants. Participants were able to lie flat in the scanner for up to 60 minutes, had no contra‐indications to MR scanning and no clinical history of neuromuscular disease. The study was approved by the Newcastle University Ethics Committee and all subjects gave written informed consent prior to enrolment.

### 2.3 Experimental set‐up

**FIGURE 2:**
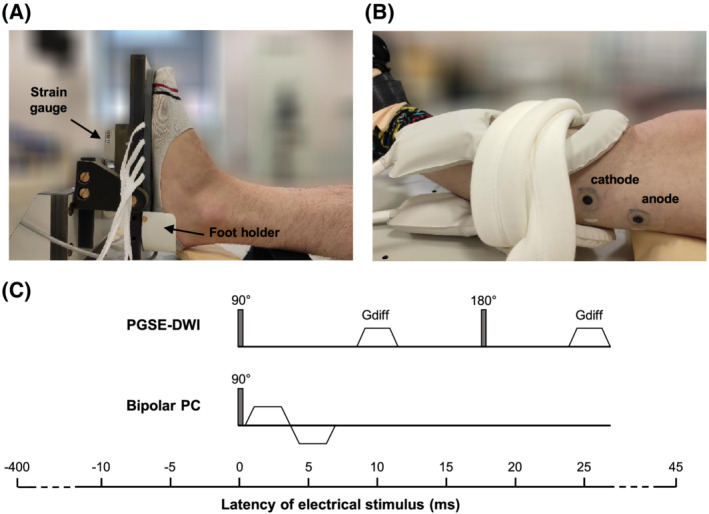
Experimental set‐up. A, Positioning of the foot in the force rig. B, Positioning of coil around the left lower leg and the placement of the stimulation electrodes for peroneal nerve stimulation, with the cathode placed distally. C, Schematic representation of the gradient waveforms for the diffusion‐weighted sequence (PGSE‐DWI) and phase contrast sequence (Bipolar PC) and the timing of the electrical stimulus pulse relative to the 90° radiofrequency pulse, in this study defined as the latency

The left lower leg of each participant was scanned in a 3 T Achieva X MR scanner (Philips Medical Systems, Best, the Netherlands). The left foot was tightly strapped in a custom‐built MR‐compatible isometric force rig containing a binocular beam load cell (capacity 60 kg, resolution 3 g; Elane, China) to measure the muscle force twitch profile (Figure 2A). This force rig was connected to a computer in the MR control room via an optical fibre. The ankle was positioned at an angle of 90‐100 and the knee extended. The knee was supported such that the lower leg muscles were not compressed and the participant's leg was fully relaxed. A pair of 10‐cm elliptical flexible surface coils or a torso coil (Philips Medical Systems) was placed above and below the left lower leg (Figure 2B).

Electrical stimulation was performed using a pair of stimulation electrodes (Cleartrace, ConMed, NY, USA) placed over the left common peroneal nerve or tibial nerve (Figure 2B). The inter‐electrode distance was 5 cm and the cathode was placed distal (Figure 2B). The stimulation electrodes were connected to a programmable stimulator (DS5, Digitimer, Fort Lauderdale, FL, USA) via MR‐compatible coaxial cables with low‐pass filters (Minicircuits, NY, USA) at the Faraday cage as previously described.^5^ A CED 1401 device (CED PLC, UK) was used to generate the stimulator drive pulse and record the data from the force rig and the scanner trigger signal. The control software for the CED 1401 was written in Microsoft Visual Basic and allowed for a variety of timing procedures.

### 2.4 Data acquisition

#### 2.4.1 Muscle twitch at I_muscle_

Data were collected during electrical stimulation, which was performed at a frequency of 1 Hz, with a bipolar square pulse (0.3 ms duration). This 1 Hz frequency was sufficiently low to allow full relaxation of motor units between stimuli. For eight volunteers, we studied the muscle twitch dynamics at a current that produced a visible muscle twitch and detectable force (I_muscle_). At this current, the muscle twitch dynamics were assessed in two ways. We applied an axial DW sequence with sensitisation along the predominant muscle fibre axis covering the left leg (FOV = 160 x 160 mm, 1.5 x 1.5 mm in‐plane resolution, 7.5 or 8 mm slice thickness, TR = 1000 ms, TE = 36 or 37 ms, Δ/δ –16.9/2.2 ms, b = 20 s/mm^2^) or both legs (FOV: 380 x 380 mm, 2 x 2 mm in‐plane resolution, TE = 38 ms). The force twitch was measured with the force rig to serve as the reference using a separate measurement recorded under identical stimulation parameters with the volunteer outside the bore to avoid the force recording being corrupted by artefacts caused by the gradient coils during scanning.

The experiment started with the collection of a set‐up DW sequence while increasing the stimulation current in coarse steps of 0.1‐2.0 mA until a clear level of contrast was observed between the stimulated and nonstimulated muscles and the muscle was twitching visibly (scan duration of ~1 minute). This current was defined as I_muscle_. In the DW set‐up scan, the stimulus pulse was given 15 ms before the 90° radiofrequency (RF) pulse (Figure 2C). We chose a latency of −15 ms because we expected the maximum contraction in the muscle at 40 ms poststimulus, and the sequence is sensitive to movement up to 27 ms after the 90° RF pulse. Subsequently, a so‐called latency DW scan was acquired (scan duration of 1.5 minutes), whereby we stimulated at I_muscle_, and altered the timing between the stimulus pulse and the 90° RF pulse in steps of 5 ms from 45 ms after the 90° RF pulse to 400 ms before the 90° RF pulse (Figure 2C; Video S1). In this way, the PGSE‐DWI gradients stepped across the muscle twitch profile, as in our theoretical model (Equations 2 to 6), and the resulting DW images could be used to generate a twitch profile.

#### 2.4.2 Single motor unit twitch at I_singleMU_

For five volunteers, we assessed twitch dynamics of single motor units with the DW sequence (the same settings as for I_muscle_) and a PC sequence (FOV = 160 x 160 mm, 1.5 x 1.5 mm in‐plane resolution, 7.5 or 8 mm slice thickness, TR = 500‐700 ms, TE = 9.7‐15.2 ms, VENC sensitivity = 1, 2 or 5 cm/s). The force twitch was not measured, because the force rig was not sensitive enough to detect the force produced by a single motor unit (~1‐15 g).^3^, ^14^

First, the set‐up DW scan and latency DW scan, as discussed for I_muscle_, were acquired. Thereafter, the stimulated muscles were delineated on the acquired latency DW images and a profile of the latency against the signal intensity was produced. From this, the latency that generated the maximum contrast (ie, with maximum signal drop) was determined and this time delay was used in the next DW scan to determine the current that activated single motor units (I_singleMU_). For this DW scan, the current was decreased in fine‐grain steps of 0.01‐0.1 mA from approximately I_muscle_ to a current level, whereby only one or two active motor units were seen on the DW images (scan duration of ~5 minutes). This current was defined as I_singleMU_. A signal void was defined as a single motor unit when it alternated as a single entity when it reached its stimulated threshold. More details on the criteria to define an active area as a single motor unit were published previously.^10^ Next, a latency DW scan (scan duration of 1.5 minutes), a latency PC scan (scan duration of 3 minutes), or both, were acquired at I_singleMU_ with a latency going from 45 to −400 ms in steps of 5 ms (Figure 2C; Video [Link], [Link]). To increase the yield of single motor units, this process was repeated one or more times per volunteer, with slightly altered distal or proximal stimulation electrode positions to recruit different motor units.

The experimental time including electrode placement and measurements for I_muscle_ and I_singleMU_ was ~30 minutes.

### 2.5 Data analysis

#### 2.5.1 DW imaging and force data analysis at I_muscle_

The stimulated muscles were delineated on the latency DW scan collected at I_muscle_ using Fiji.^15^ For peroneal nerve stimulation, the region of interest (ROI) contained the tibialis anterior (TA), extensor digitorum longus (EDL) and peroneus longus (PL) muscles. For tibial nerve stimulation, the ROI contained the gastrocnemius medialis, gastrocnemius lateralis and soleus (SOL) muscles. The average signal intensity in the ROI was determined per latency step to create the latency DW signal. The signal was normalised to baseline using the average signal intensity in the first five latency steps, since these time points showed no signal drop yet. From the latency DW signal, we determined the width of the first signal drop and its percentage signal drop (Figure 1B). The width was automatically calculated as the latency at the start of the signal drop minus the latency at the end of the signal drop. The start point was defined as one time point before the signal dropped below 95% of baseline signal, and the end point was the time point with maximum signal in the period between the first and second signal drop.

The force data were filtered with a 100‐Hz low‐pass filter to remove high frequency noise and were subsequently baseline‐corrected by subtracting the average force from 10 ms before to 5 ms after the stimulus pulse. The force contraction time was determined as the time from the start of the twitch to the time at maximum displacement (Figure 1B). The start of the twitch was defined as the first time that the force signal was larger than 5 times the baseline standard deviation.

Furthermore, the experimental force data were used as input for the theoretical model, and the corresponding net magnetisation was simulated with the PGSE‐DWI gradient waveform and the contraction model of 3%, 5% and 7% asymmetric compaction and no translation.

#### 2.5.2 DW imaging and PC imaging analysis at I_singleMU_

Using Fiji, each active motor unit was manually delineated on the DW scans, because on those scans the edges of the motor unit were sharpest.^15^ A voxel was included in a motor unit ROI if there was a visually clear signal drop. If a single motor unit latency DW scan was acquired, the ROI was drawn on this latency DW scan, otherwise the ROI was drawn on the DW scan used to estimate I_singleMU_. Then the average signal in the DW and PC images within this ROI were determined per latency time point.

For the DW scan, the width of first signal drop was determined as described for I_muscle_, with the difference that the end of the peak was the time point at which the signal returned to more than 95% of the baseline signal. This is because no clear second peak was observed in the single motor unit DW latency scans.

For the PC images, the velocity contained an offset, which can be attributed to the presence of B0 eddy currents, concomitant fields and gradient nonlinearity.^16^ The offset was removed in two steps (see the supporting material: Methods – Removing offset in phase contrast images and Figure S1 for more information). The contraction time using the PC data was calculated as the difference between the time points at the start of displacement and at maximum displacement. The start of displacement was calculated from the velocity signal being one time point before the velocity increased by 10%.

The spatial distribution of the DW and PC changes were compared by plotting the voxels in which changes in the DW signal and velocity were seen. For the DW changes, a difference map was created by subtracting the dynamic with the maximal signal drop from the average of the first five dynamics, which was then normalised to its maximum (Figure S2).^10^ Voxels were defined as showing a DW change if their value in the normalised difference map was larger than 0.5; a value of 0.5 was previously validated.^10^ For PC, voxels were included if the velocity was larger than 0.15 cm/s, the standard deviation over nonactive muscle (Figure S2). This phase analysis was performed on the dynamic showing the maximum positive velocity.

### 2.6 Statistical analysis

Statistical analysis was performed with SPSS Statistics version 25 (SPSS, Chicago, IL, USA). For the simulated data, a linear regression model was applied between the contraction time of the theoretical twitch and the contraction time assessed from the simulated net magnetisation and phase signal, to both examine the relationship between the true and measured timing and assess the impact of the imaging acquisition window parameters. Furthermore, the simulated percentage signal drop and twitch contraction time were fitted with an exponential function. For the experimental data, measurements were compared with the intraclass correlation coefficient (ICC), based on single measurements, a two‐way mixed effect model and consistency. Significance was set at *P* less than .05. Data are presented as mean ± SD unless otherwise stated.

## 3 RESULTS

### 3.1 Theoretical model

#### 3.1.1 Effect of twitch contraction time

**FIGURE 3:**
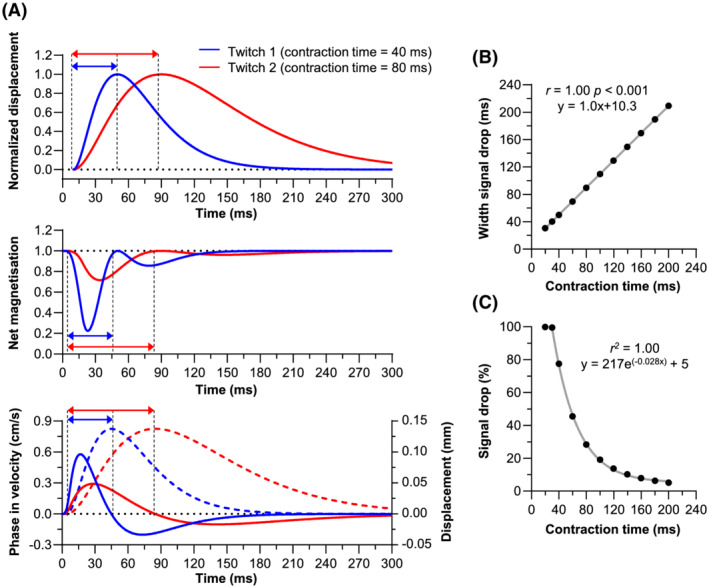
Simulation results. Effect of twitch contraction time for a contraction with 5% asymmetric compaction and no translation. A, Simulation results for a theoretical twitch with contraction times of 40 ms (blue) and 80 ms (red). Top, Theoretical twitch, with arrows representing the contraction time. Middle, Simulated net magnetisation, with the arrows representing the width of the first signal drop. Bottom, Simulated cumulative phase, expressed in velocity (solid), and its integral (dashed), with the arrows representing the contraction time of the integrated phase signal. B, Contraction time vs. width of the first signal drop in the net magnetisation. C, Contraction time vs. percentage signal drop in net magnetisation. Note: at 30 ms contraction the maximum signal drop (100%) was reached, wherefore no additional signal drop is seen at 20 ms compared with 30 ms contraction time. Therefore, the 20 ms contraction time was not included in the exponential fit

During asymmetric compaction, the modelled net DW magnetisation exhibited two consecutive drops with the PGSE‐DWI gradient waveform, while the cumulative phase showed first a positive peak followed by a negative peak using the Bipolar PC waveform (Figure 3A). These two phases coincide with the contraction and relaxation in the muscle twitch. The theoretical twitch contraction time was linearly related to the width of the first signal drop in net magnetisation and the integrated phase contraction time. Both had a slope of 1.0, and the introduced offset between the input twitch contraction time and measured contraction time was 10.3 ms for PGSE‐DWI and 4.5 ms for Bipolar PC (Figure 3B, Figure S3A). Furthermore, the twitch contraction time showed a nonlinear relationship with the percentage drop in net magnetisation and maximum positive velocity, which could be fitted by a negative exponential function (r^2^ = 1.000 and r^2^ = 0.993, respectively; Figure 3C, Figure S3B).

#### 3.1.2 Effect of percentage compaction and translation

**FIGURE 4:**
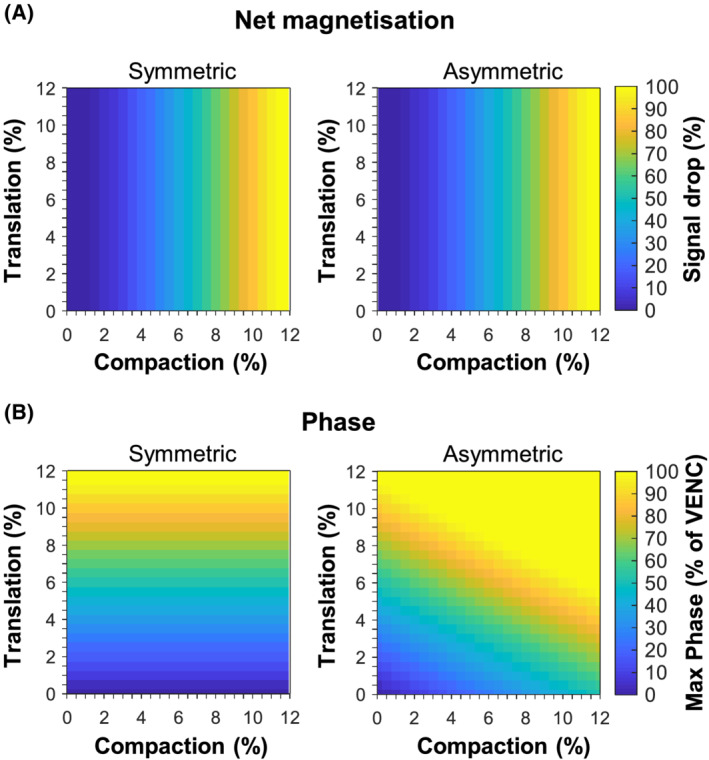
Simulation results. Effect of percentage compaction and translation. A, Percentage signal drop in net magnetisation for symmetric compaction (left) and asymmetric compaction (right). B, Maximum phase in the cumulative phase expressed as percentage of velocity‐encoding sensitivity (VENC; 1.3 cm/s) for symmetric compaction (left) and asymmetric compaction (right)

Varying the percentage compaction and translation showed that the percentage drop in the net magnetisation increased with more compaction, but was not influenced by translation (Figure 4A). There was no difference between symmetric and asymmetric compaction on the net magnetisation. The minimum value of the simulated net magnetisation reached 0 (ie, total signal dephasing) at 11.5% compaction.

For symmetric compaction and translation (situation 4), the maximum velocity determined from the cumulative phase was linearly related with the percentage translation (more displacement in the same amount of time) and was independent of compaction (Figure 4B, Figure S4). The use of the Bipolar PC gradient with a VENC sensitivity of 1.3 cm/s led to aliasing at 11.5% translation. In the case of asymmetric compaction and translation (situation 5), the maximum velocity showed a linear relationship with compaction and translation, whereby the moment of aliasing depended on the combination of percentage compaction and percentage translation (Figure 4B, Figure S4).

### 3.2 Experimental data

#### 3.2.1 Participants

**FIGURE 5:**
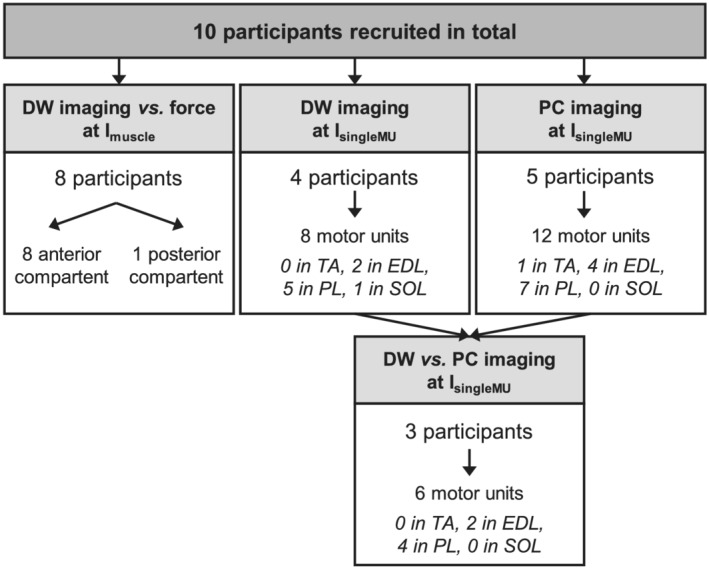
Study flowchart. The flowchart reflects the number of participants and motor units studied at a stimulation current producing a visible muscle twitch (I_muscle_) and a current activating single motor units (I_singleMU_). DW, diffusion‐weighted; EDL, extensor digitorum longus; PC, phase contrast; PL, peroneus longus; SOL, soleus; TA, tibialis anterior

Ten participants (six males) were recruited for this study, with an average age of 30 ± 10 (range: 20‐48) years. From eight of these volunteers (five males, age: 33 ± 10 years), a muscle twitch force and DW latency scan of the anterior muscle compartment was acquired by stimulating the peroneal nerve at I_muscle_ (Figure 5). In one of them, the posterior muscle compartment was also assessed by stimulating the posterior tibial nerve. Single motor unit latency DW scans at I_singleMU_ were acquired in four participants (two males, 33 ± 10 years), latency PC scans in five participants (three males, 29 ± 11 years) and both latency DW and latency PC scans in three of these participants (two males, 34 ± 12 years).

We were able to analyse eight motor units from the latency DW scans and 12 motor units from the latency PC scans, of which six were imaged with both a DW and a PC sequence. These motor units were found in the TA, EDL, PL and SOL (Figure 5).

#### 3.2.2 DW imaging and force data at I_muscle_

**FIGURE 6:**
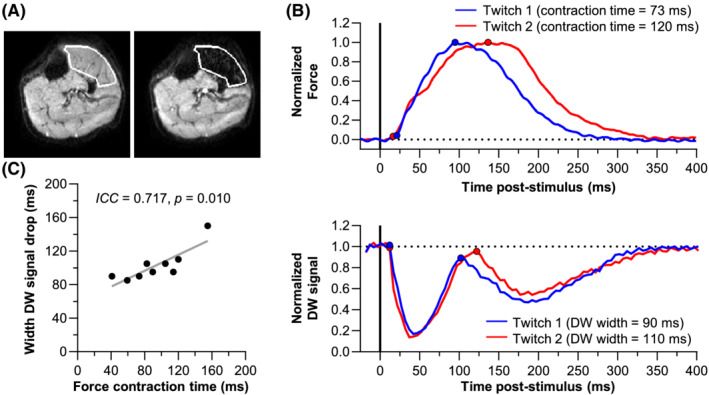
Force and diffusion‐weighted (DW) signal at the current level producing a visible muscle twitch (I_muscle_). A, Example DW images with the anterior muscle compartment delineated. The left image represents a latency with the diffusion gradients placed 18 ms prestimulus showing no signal drop, and the right image was acquired 42 ms poststimulus, where anterior compartment muscles reached maximum drop in signal. B, Typical examples of normalised force twitch profiles (top) and their corresponding DW signal changes (bottom) of the anterior compartment muscles of two volunteers. The start of the muscle contraction and maximum displacement in the force signal and the start and end of the first DW signal drop are depicted with a circle. Maximum contrast was reached at 30 to 45 ms poststimulus. This would equal an experimental set latency of −3 to −18 ms (ie, a stimulus pulse given 3‐18 ms before 90° RF pulse); the poststimulus time was calculated as: −1 × (experimental latency ‐ the 27 ms between the RF pulse and the end of the diffusion sensitive period [until the end of the diffusion gradients]). In other words, if the stimulus is given at the 90° RF pulse, the corresponding DW signal is depicted at 27 ms poststimulus (Figure 2C). C, Force contraction time vs. width of the first signal drop in the DW signal

The mean stimulus current I_muscle_ was 13.4 ± 6.1 mA. This current made the stimulated muscle fully black on the DW images (Figure 6A and Video S1). Furthermore, it produced an observable muscle force twitch (Figure 6B), with an average contraction time of 93 ± 34 ms (Table 1). The latency DW signal in the stimulated muscles showed two consecutive drops, in line with the theoretical model, and reflecting the contraction and relaxation of muscle fibres (Figure 6B). The width of the first DW signal drop was 103 ± 20 ms (Table 1) and was comparable with the force contraction time (ICC = 0.717, *P* = .010; Figure 6C).

**TABLE 1:** Twitch properties assessed with force, diffusion‐weighted (DW) imaging, and phase contrast (PC) imaging

At I_muscle_	N	
Force contraction time (ms)	9	93 ± 34 (41‐155)
Width first DWI signal drop (ms)	9	103 ± 20 (85‐150)

At I_singleMU_		
Width first DW signal drop (ms)	8	75 ± 13 (55‐95)
PC contraction time (ms)	12	81 ± 15 (55‐100)
Maximum positive velocity (cm/s)	12	0.55 ± 0.26 (0.21‐1.04)
Maximum negative velocity (cm/s)	12	−0.22 ± 0.10 (−0.44 to −0.10)
Displacement (mm)	12	0.20 ± 0.10 (0.05‐0.37)

Data are presented as mean ± SD with the range given in parentheses

The polynomial fit of the experimental force twitch was used as input for the theoretical model to further explore the mechanism of signal change. The results are described in detail in the supporting information. In brief, the width of the first drop in the DW signal of the simulated and measured latency signal were comparable (ICC = 0.718, *P* = .010; Figure S5A). Furthermore, this analysis indicates that at least 7% compaction was needed to achieve a percentage drop in the simulated net magnetisation in the same range as the percentage drop in the measured DW signal (Figure S5B).

#### 3.2.3 DW imaging and PC imaging at I_singleMU_

**FIGURE 7:**
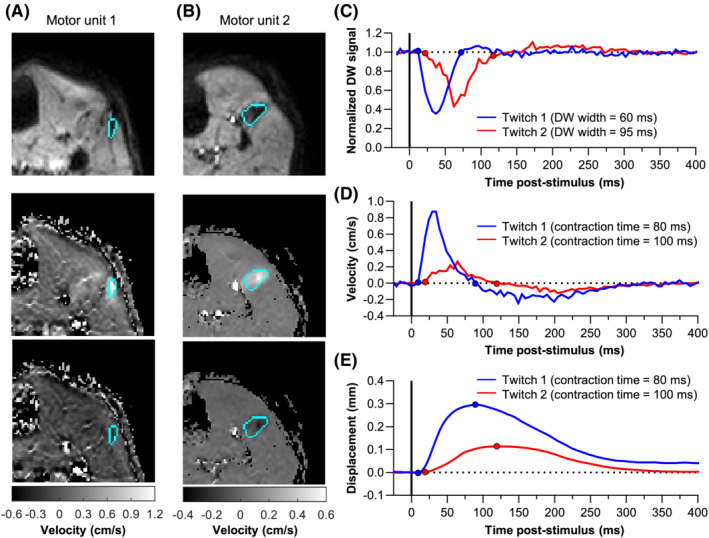
Typical examples of diffusion‐weighted (DW) imaging and phase contrast (PC) imaging at a current level activating a single motor unit (I_singleMU_). A and B, Two typical examples of a motor unit (delineated in light blue) on a DW image (top) and PC image (middle/bottom). The DW image is shown at the time point that the DW signal reaches its maximum signal drop. The PC images are shown for the time point that the velocity was maximum (middle) and minimum (bottom). C, Normalised DW signal intensity displayed against the time poststimulus for the motor units shown in A and B. The start and end of the first signal drop in the DW signal are depicted with a circle. D, Velocity measured with the PC sequence. E, Displacement (integrated velocity signal). The start and end of the contraction period are depicted in circles. The poststimulus time was calculated as: −1 × (experimental latency ‐ the period the sequence is sensitive to movement [until the end of the diffusion gradients]); the movement sensitive time is 27 ms for the DW signal, 4.22 ms for the phase signal of motor unit 1 (velocity‐encoding sensitivity [VENC] = 5 cm/s) and 9.13 ms for motor unit 2 (VENC = 1 cm/s). In other words, if the stimulus is given at the 90° RF pulse, this is for the DW signal depicted at 27 ms poststimulus

The mean stimulus current I_singleMU_ was 8.6 ± 3.3 mA. During the contraction, the activated motor units became black in the DW images and showed a positive velocity on the phase images (Figure 7A,B and Video 9, 10). During the relaxation, the velocity on the phase images became negative in all studied motor units (Figure 7A,B). Interestingly, the relaxation phase did not lead to a second signal drop in the latency DW signal, instead the latency DW signal sometimes showed a very small increase in signal intensity (Figure 7C). The latency PC signal showed two peaks, first a velocity in the positive direction (average: 0.55 ± 0.26 cm/s) followed by a velocity in the opposite negative direction (average: −0.22 ± 0.10 cm/s), as predicted by the theoretical model (Figure 7D, Table 1). Integrating this signal gives the displacement, representing the muscle twitch, resulting in an average displacement of 0.20 ± 0.10 mm (Figure 7E). The average width of the first DW signal drop was 75 ± 13 (range 55‐95) ms and the PC contraction time was 81 ± 15 (range: 55‐100) ms (Table 1). The contraction times of the six motor units measured with both DW and PC imaging were comparable (ICC = 0.925, *P* = .001; Figure S6).

**FIGURE 8:**
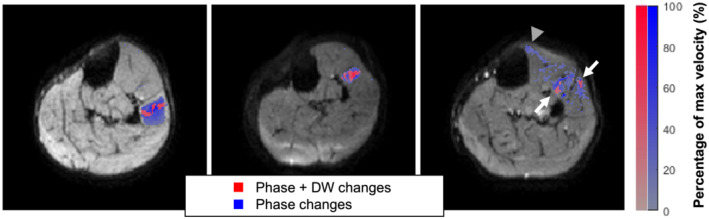
Spatial distribution of the diffusion‐weighted (DW) and phase changes. Three examples are shown from left to right with the following colour coding. Red: voxels with phase and DW changes. Blue: voxels with only phase changes. All three examples show areas with DW changes and phase changes in the middle of the motor unit, whereby the phase changes extend further through the muscle than the DW changes. No voxels were observed with only DW changes. In the right example, two motor units were detected with the DW signal (white arrows) and a possible third can only be detected by the phase (grey arrowhead)

Figure 8 shows the overlay of the DW changes and phase change. This reveals that DW changes occurred in a small area, while the phase changes extended much further through the muscle (left and middle). For the example on the right, the DW images showed two areas of activity (red), while in the phase images there also seemed to be a third area of activity (blue).

## 4 DISCUSSION

This study investigated the effect of muscle fibre contraction on the DW MUMRI and PC MUMRI images by combining a computational model and experimental data. The modelling shows that the signal attenuations in DW magnitude images are only sensitive to dephasing and not to general phase offsets. This means that they are sensitive to compaction, but cannot distinguish between symmetric or asymmetric compaction, and that they are insensitive to translation (as long as it translates with constant velocity). Opposite to this, the model shows that the phase signal in PC images is only sensitive to absolute phase differences and can therefore detect asymmetric compaction and translation. This is clearly reflected in our experimental data, where DW changes induced by a single motor unit activation are localised and coincide with phase changes (and reveal the direct action of the motor unit). By contrast, phase changes are more widespread through the muscle, even with a highly conservative threshold of 0.15 cm/s, and they are induced by bulk movement. Therefore, phase changes also reveal the effect of active motor unit contraction on physically coupled surrounding inactive muscle fibres.

In our simulation model the gradient waveform was moved along the twitch profile, which showed that muscle fibre contraction and relaxation are reflected in the net magnetisation as two consecutive signal drops, and in the phase signal as a positive velocity peak followed by a negative velocity peak. This biphasic response was also observed in the experimentally acquired latency DW signal and PC signal. The width of the first signal drop in the DW signal was comparable with the force contraction time, and could be reproduced with the model. This confirms that the latency DW and PC signal profile produced by shifting the timing between the stimulus pulse and imaging acquisition window reflect the muscle twitch and can measure single motor unit twitch profiles.

In our single motor unit data, the average contraction time was 75 (range: 55‐95) ms with a DW sequence and 81 (55‐100) ms with a PC sequence. These contraction times have an offset caused by the convolution of the true response and the response of the imaging acquisition window (PGSE‐DWI: 10.3 ms and Bipolar PC: 4.5 ms). This offset will depend on the diffusion time (Δ). If we correct for this offset, the DW‐ and PC‐determined contraction times are close to the contraction times previously reported for motor units in the lower leg: 61 (range 40‐90) ms in the TA, 48 (38‐67) ms in the EDL, 76 (40‐110) ms in the gastrocnemius medialis and 127 (64‐251) ms in the SOL.^14^, ^17^, ^18^, ^19^ This offset also explains the slightly longer average DW contraction time (103 [range 85‐150] ms) compared with the average force contraction time (93 [41‐155] ms) for measurements at I_muscle_. This suggests that MUMRI can accurately and noninvasively assess the relative contraction time of single motor units and whole muscle twitches.

During contraction, the signal attenuation in the DW signal and the PC‐measured positive velocity increased with more displacement in the same amount of time, that is, larger percentage compaction and translation, and a higher contractile rate. The computational model suggests that the contracting muscle fibres in the experimental measurements were compacting by at least 7%. This is not unreasonable given that muscle fascicles in the TA can contract by up to 12% during walking, and that the tendon and aponeurosis can show at least 1‐cm elongation during isometric contraction.^20^, ^21^, ^22^ However, the 7% compaction should be interpreted with care, because the percentage compaction during the experiment was not controlled for and the 7% estimated depends on the assumptions in the model. The simulated contraction is a 1D approximation and does not capture the complexity of 3D muscle deformation and represents a simple uncoupled system that does not include elastic elements like the tendon.

During relaxation, the DW signal displayed a second signal drop with whole muscle activation, but not when a single motor unit was active. Instead, sometimes a slight signal increase was observed during relaxation. The absence of the second signal drop could be due to the slower relaxation velocity (~0.2 cm/s) compared with the contractile velocity (~0.5 cm/s). According to the simulation, twitches with a maximum contractile velocity of 0.2 cm/s exhibited only up to 10% of signal loss. Furthermore, the diffusion sequence was sensitive to diffusion only in the slice direction, while a motor unit twitch is a complex 3D process with muscle cells changing shape and angle during contraction and relaxation. If the magnitude of incoherent motion in the slice direction was lower during relaxation compared with contraction then there will be less of a reduction in the DW signal. This is supported by observations showing that the ratio between the first and second DW signal drop depends on the diffusion direction (unpublished preliminary data). However, these arguments do not explain why the signal would increase during relaxation. A DW signal can increase due to a reduced diffusion coefficient, increased proton density or increased T2 relaxation time (T2 shine‐through effect), but it is unlikely that one of these parameters alone can explain our observations.^23^, ^24^ With the used b‐value of 20 s/mm^2^, a reduced diffusion coefficient can increase the signal intensity in muscle with maximal ~3%, and a sudden 10% increase in proton density during relaxation is unlikely. Repeated stimulation can lead to increased T2 relaxation time in the muscle, however, this needs to elevate with ~3 ms to obtain a signal increase of 10%, and with the stimulus frequency used in our study no changes in T2 relaxation time were observed.^25^, ^26^ Furthermore, this effect does not disappear instantaneously after exercise, while in our data the signal increase disappeared during the last few stimulations and remained absent directly after stimulation. Another potential explanation is T1 saturation; in the case of 5% compaction, the signal would increase by 4.8% due to new protons moving into the imaging slice that were not excited in the previous TR. At the moment, we do not fully understand the absence of a second signal drop during single motor unit relaxation, but this does not affect the ability to determine the motor unit type, since this depends on the contraction time.

Previous studies assessing the contractile properties of single motor units used intraneural or intramuscular needle stimulation, requiring extremely sensitive force transducers, and it was hard to validate if the twitch originated from a single motor unit.^14^, ^18^, ^19^ MUMRI has the advantage that motor units are stimulated via surface electrodes and that the imaging gives a 2D image of the stimulated motor unit. This image can be used to detect alternation, to verify that only a single motor unit was activated.^10^ MUMRI can thus measure time dynamics, contractile velocity and the motor unit size, which are three characteristics that discriminate fast and slow twitch motor units,^2^ all in a clinical feasible scan time of 30 minutes. Therefore, MUMRI could potentially be used to study the preferential loss of fast twitch motor units, and the resulting transition of fast twitch muscle fibres to slow twitch muscle fibres and increased motor unit sizes that occur in ALS and sarcopenia.^27‐31^ Currently, this has only been done in mice or via muscle biopsies in humans.^27‐31^ To make such a study possible, we first need to validate that MUMRI can discriminate between slow twitch and fast twitch muscle fibres, for example, by comparing single motor unit twitch properties in muscles comprising predominantly fast twitch fibres and muscles comprising predominantly slow twitch fibres. Furthermore, the technique should be able to sample multiple motor units per muscle, especially since motor unit recruitment at low levels of electrical stimulation will bias us towards the large slow twitch motor units.^27^, ^28^ Currently, our technique is limited to one or two motor units per acquisition, although this can be increased by repeating the acquisition with an altered electrode position.

We analysed the magnitude image of the DW sequence and the phase image of the PC sequence. Both can be used to extract the contraction time of single motor units and have their own advantages and disadvantages. The phase image outputs the velocity of the twitch, which can be integrated to extract the twitch profile. On the other hand, the magnitude image gives the best estimate of the motor unit's spatial extent, because it detects only the active part of the motor unit; previous work showed that the single motor unit size was in concordance with scanning electromyography.^10^ The phase signal also detects passively translating fibres and will therefore probably overestimate the motor unit size. The DW sequence and PC sequence are both similarly sensitive to velocity (phase change) and incoherent motion (magnitude change), although they are traditionally described by b‐value and VENC sensitivity, respectively. Therefore, an obvious issue is if one sequence could be applied, whereby the phase image is used to assess the velocity of the muscle twitch and the magnitude image is used to estimate the spatial extent of the motor unit. Our work shows that the VENC sensitivity should be ~1 to 2 cm/s when studying single motor units. For the PC sequence, this would equal a b‐value of ~5 s/mm^2^. Inspection of the magnitude images reveals that this 5 s/mm^2^ is too low to quantify motor unit activity in the magnitude signal. Conversely, higher b‐values, for example, 20 s/mm^2^, as used in the PGSE‐DWI sequence that give sufficient contrast in the magnitude image, come at the cost of lower VENC sensitivity, which would induce aliasing in the phase signal.

The computational simulations and the experimental measurements have a few limitations. The muscle fibre contraction was simulated in 1D with the assumption that the muscle fibre contraction was in the same direction as the diffusion encoding. However, in vivo, the fibre contraction is likely to take place at a different angle than the diffusion encoding, and there will be 3D deformations; the muscle fibres become thicker when they contract and the pennation angle may change. There is no doubt that this will affect the signal attenuation in the DW signal, but it is difficult to predict in what way. This issue could potentially be dealt with using an isotropic tensor‐encoding diffusion sequence.^29^ Furthermore, we assumed that all points of magnetisation remained in the slice. If points moved out of the slice this would give additional signal loss, but with the low level of compaction and translation used in our simulations, this effect will be minimal. The experimental work was performed retrospectively on a limited number of participants. Despite these low numbers, we found the relationship between the width of the first signal drop in the DW signal and the twitch contraction time, but more participants, and especially assessments of both known slow twitch muscles and known fast twitch muscles, are needed to confirm this relationship. Regarding the 14 studied single motor units, the reported twitch contraction times and velocities cannot serve as a representative description of lower leg motor units, but only as a first indication that MUMRI is able to measure the twitch profile reliably. The DW single motor latency scans were always acquired before the PC single motor unit latency scans, but there is no indication that at the low current we used, the order of the scans, for example, via fatigue, influenced the results. Furthermore, our work was mainly limited to the anterior compartment muscles because their muscle fibres run mostly parallel and contract along the diffusion‐sensitive direction, and the muscles are easily activated with surface electrodes via the superficial laying innervating nerve. The lower leg's posterior compartment was more difficult to activate because the posterior tibial nerve lies deeper in the popliteal fossa, and therefore requires higher stimulation strengths, making it less comfortable for the participant.

In conclusion, we showed that the primary contrast mechanism for the signal voids in DW images of active skeletal muscle is compaction, which detects the active part of the motor unit. The phase changes were more widespread, because they also reflect translation of muscle tissue that is passively pulled along. The DW signal attenuations and phase changes are dependent on the magnitude of the contraction and the contractile properties of the motor unit, making it possible to measure the motor unit's twitch profile by altering the timing of stimulation in relation to the imaging acquisition window. This makes MUMRI a promising tool with which to assess motor unit composition in vivo.

## FUNDING INFORMATION

This work was supported by the Rubicon research programme (project number: 452183002) of the Dutch Research Council (Nederlandse Organisatie voor Wetenschappelijk Onderzoek (NWO)), by the Medical Research Council Confidence in Concept (CiC) award (Newcastle University study number 1621/7484/2018), by Muscular Dystrophy UK (grant number: 18GROPG36–0246‐1) and the National institute for Health Research (NIHR) Newcastle Biomedical Research Centre. The NIHR Newcastle BRC is a partnership between Newcastle Hospitals NHS Foundation Trust and Newcastle University, funded by the NIHR. This paper presents independent research funded and supported by the NIHR Newcastle BRC. The views expressed are those of the authors and not necessarily those of the NIHR or the Department of Health and Social Care.

## Supporting information


**Figure S1:** Supporting Information

**Figure S2:** Supporting Information

**Figure S3:** Supporting Information

**Figure S4:** Supporting Information

**Figure S5:** Supporting Information

**Figure S6** Supporting Information

**Figure S1:** Phase error correction. Automatic detection of non‐active muscle. A) Original phase image whereby non‐active muscle displays a velocity off‐set (higher than 0 cm/s). B) Histogram of phase values of all voxels in the phase image of A. C) Selected non‐active muscle overlaid in red on the phase image of A. D) Baseline corrected phase image showing that non‐active muscle now has a velocity of 0 cm/s.
**Figure S2:** Schematic overview of voxel‐by‐voxel assessment of significant diffusion weighted (DW) and phase changes for the 2D overlay. A) Overview for detection of significant DW changes. B) Overview for detection of significant phase (velocity) changes.
**Figure S3:** Simulation results. Effect of twitch contraction time on cumulative phase signal. A) Phase contraction vs twitch contraction time. B) Maximum phase change in velocity vs. contraction time.
**Figure S4:** Simulation results. Effect of percentage compaction and translation for asymmetric compaction. A) Percentage signal drop in net magnetisation versus percentage translation showing that percentage signal drop is independent of translation. Each line represents a percentage compaction. B) Percentage signal drop in net magnetisation versus percentage compaction. Each line represents a percentage translation showing a sigmoidal relation between percentage compaction and percentage signal drop, whereby the signal reached 100% signal drop at 11.5% compaction. C) Maximum phase expressed as percentage of VENC (1.3 cm/s) versus percentage translation showing a linear relation between percentage translation and maximum phase. D) Maximum phase expressed as percentage of VENC (1.3 cm/s) versus percentage compaction showing a linear relation between percentage compaction and maximum phase.
**Figure S5:** Comparison of the experimental diffusion weighted (DW) signal changes and the simulated magnetisation changes using the measured force as model input. A) Width of the first DW signal drop. B) Percentage signal drop for three levels of asymmetric compaction, 3%, 5% and 7%, and no translation, with a linear regression line for the 7% compaction.
**Figure S6:** Correlation graph of phase contrast contraction time *vs.* width of the first diffusion weighted (DW) signal drop. Data were acquired at the stimulation current I_singleMU_ that activated single motor units.
**Video S1:** Latency diffusion weighted (DW) scan at stimulation current producing a visible muscle twitch (I_muscle_)
**Video S2:** Latency diffusion weighted (DW) scan at a stimulation current activing a single motor unit (I_singleMU_)
**Video S3:** Latency phase contrast (PC) scan at a stimulating current activating a single motor unit (I_singleMU_)

**Video S1:** Supporting Information

**Video S2:** Supporting Information

**Video S3:** Supporting Information

## Data Availability

The data that support the findings of this study are available from the corresponding author upon reasonable request.

## References

[1] Garg N , Park SB , Vucic S , et al. Differentiating lower motor neuron syndromes. J Neurol Neurosurg Psychiatry. 2017;88(6):474‐483. 28003344 10.1136/jnnp-2016-313526 PMC5529975

[2] Buchthal F , Schmalbruch H . Motor unit of mammalian muscle. Physiol Rev. 1980;60(1):90‐142. 6766557 10.1152/physrev.1980.60.1.90

[3] English AW , Wolf SL . The motor unit. Phys Ther. 1982;62(12):1763‐1772. 6216490 10.1093/ptj/62.12.1763

[4] Heckman CJ , Enoka RM . Motor Unit. Compr Physiol. 2012;2:2629‐2682. 23720261 10.1002/cphy.c100087

[5] Whittaker RG , Porcari P , Braz L , Williams TL , Schofield IS , Blamire AM . Functional magnetic resonance imaging of human motor unit fasciculation in amyotrophic lateral sclerosis. Ann Neurol. 2019;85(3):455‐459. 30688362 10.1002/ana.25422

[6] Steidle G , Schick F . Addressing spontaneous signal voids in repetitive single‐shot DWI of musculature: Spatial and temporal patterns in the calves of healthy volunteers and consideration of unintended muscle activities as underlying mechanism. NMR Biomed. 2015;28(7):801‐810. 25943431 10.1002/nbm.3311

[7] Schwartz M , Martirosian P , Steidle G , et al. Volumetric assessment of spontaneous mechanical activities by simultaneous multi‐slice MRI techniques with correlation to muscle fiber orientation. NMR Biomed. 2018;31(11):e3959. 30067885 10.1002/nbm.3959

[8] Schwartz M , Steidle G , Martirosian P , et al. Spontaneous mechanical and electrical activities of human calf musculature at rest assessed by repetitive single‐shot diffusion‐weighted MRI and simultaneous surface electromyography. Magn Reson Med. 2018;79(5):2784‐2794. 28921633 10.1002/mrm.26921

[9] Karampinos DC , Banerjee S , King KF , Link TM , Majumdar S . Considerations in high‐resolution skeletal muscle diffusion tensor imaging using single‐shot echo planar imaging with stimulated‐echo preparation and sensitivity encoding. NMR Biomed. 2012;25(5):766‐778. 22081519 10.1002/nbm.1791 PMC3299872

[10] Birkbeck MG , Heskamp L , Schofield IS , Blamire AM , Whittaker RG . Non‐invasive imaging of single human motor units. Clin Neurophysiol. 2020;131(6):1399‐1406. 32122767 10.1016/j.clinph.2020.02.004 PMC7208543

[11] Nayler GL , Firmin DN , Longmore DB . Blood flow imaging by cine magnetic resonance. J Comput Assist Tomogr. 10(5):715‐722. 3528245 10.1097/00004728-198609000-00001

[12] Pelc NJ , Herfkens RJ , Shimakawa A , Enzmann DR . Phase contrast cine magnetic resonance imaging. Magn Reson Q. 1991;7(4):229‐254. 1790111

[13] Bélanger AY , McComas AJ . A comparison of contractile properties in human arm and leg muscles. Eur J Appl Physiol Occup Physiol. 1985;54(3):326‐330. 4065119 10.1007/BF00426154

[14] Garnett RA , O'Donovan MJ , Stephens JA , Taylor A . Motor unit organization of human medial gastrocnemius. J Physiol. 1979;287(1):33‐43. 430414 10.1113/jphysiol.1979.sp012643 PMC1281479

[15] Schindelin J , Arganda‐Carreras I , Frise E , et al. Fiji: an open‐source platform for biological‐image analysis. Nat Methods. 2012;9(7):676‐682. 22743772 10.1038/nmeth.2019 PMC3855844

[16] Gatehouse PD , Rolf MP , Graves MJ , et al. Flow measurement by cardiovascular magnetic resonance: a multi‐centre multi‐vendor study of background phase offset errors that can compromise the accuracy of derived regurgitant or shunt flow measurements. J Cardiovasc Magn Reson. 2010;12(1):5 10.1186/1532-429X-12-5 20074359 PMC2818657

[17] Sanchez GN , Sinha S , Liske H , et al. In vivo imaging of human sarcomere twitch dynamics in individual motor units. Neuron. 2015;88(6):1109‐1120. 26687220 10.1016/j.neuron.2015.11.022 PMC5920519

[18] Leitch M , Macefield VG . Comparison of contractile responses of single human motor units in the toe extensors during unloaded and loaded isotonic and isometric conditions. J Neurophysiol. 2015;114(2):1083‐1089. 26041824 10.1152/jn.00121.2015 PMC4725102

[19] Andreassen S , Arendt‐Nielsen L . Muscle fibre conduction velocity in motor units of the human anterior tibial muscle: a new size principle parameter. J Physiol. 1987;391(1):561‐571. 3443957 10.1113/jphysiol.1987.sp016756 PMC1192232

[20] Maganaris CN , Paul JP . In vivo human tendinous tissue stretch upon maximum muscle force generation. J Biomech. 2000;33(11):1453‐1459. 10940404 10.1016/s0021-9290(00)00099-3

[21] Maganaris CN , Paul JP . Load‐elongation characteristics of in vivo human tendon and aponeurosis. J Exp Biol. 2000;203(Pt 4):751‐756. 10648216 10.1242/jeb.203.4.751

[22] Chleboun GS , Busic AB , Graham KK , Stuckey HA . Fascicle length change of the human tibialis anterior and vastus lateralis during walking. J Orthop Sport Phys Ther. 2007;37(7):372‐379. 10.2519/jospt.2007.2440 17710906

[23] Dietrich O , Biffar A , Baur‐Melnyk A , Reiser MF . Technical aspects of MR diffusion imaging of the body. Eur J Radiol. 2010;76(3):314‐322. 20299172 10.1016/j.ejrad.2010.02.018

[24] Bammer R . Basic principles of diffusion‐weighted imaging. Eur J Radiol. 2003;45(3):169‐184. 12595101 10.1016/s0720-048x(02)00303-0

[25] Prompers JJ , Jeneson JAL , Drost MR , Oomens CCW , Strijkers GJ , Nicolay K . Dynamic MRS and MRI of skeletal muscle function and biomechanics. NMR Biomed. 2006;19(7):927‐953. 17075956 10.1002/nbm.1095

[26] Varghese J , Scandling D , Joshi R , et al. Rapid assessment of quantitative T 1, T 2 and T 2 * in lower extremity muscles in response to maximal treadmill exercise. NMR Biomed. 2015;28(8):998‐1008. 26123219 10.1002/nbm.3332 PMC4524289

[27] Singh K , Richmond FJ , Loeb GE . Recruitment properties of intramuscular and nerve‐trunk stimulating electrodes. IEEE Trans Rehabil Eng. 2000;8(3):276‐285. 11001507

[28] Grill WM . Model‐based analysis and design of waveforms for efficient neural stimulation. Prog Brain Res. 2015;222(1):147‐162. 26541380 10.1016/bs.pbr.2015.07.031 PMC4772858

[29] Szczepankiewicz F , Sjölund J , Ståhlberg F , Lätt J , Nilsson M . Tensor‐valued diffusion encoding for diffusional variance decomposition (DIVIDE): Technical feasibility in clinical MRI systems. PLoS ONE. 2019;14(3):e0214238. 30921381 10.1371/journal.pone.0214238 PMC6438503

