# Supplementary figures and images for: The muscle twitch profile assessed with motor unit magnetic resonance imaging
Source: NMR Biomed. 2021 Jan 6;34(3):e4466. doi: 10.1002/nbm.4466 (PMC7900994; doi:10.1002/nbm.4466)

**A**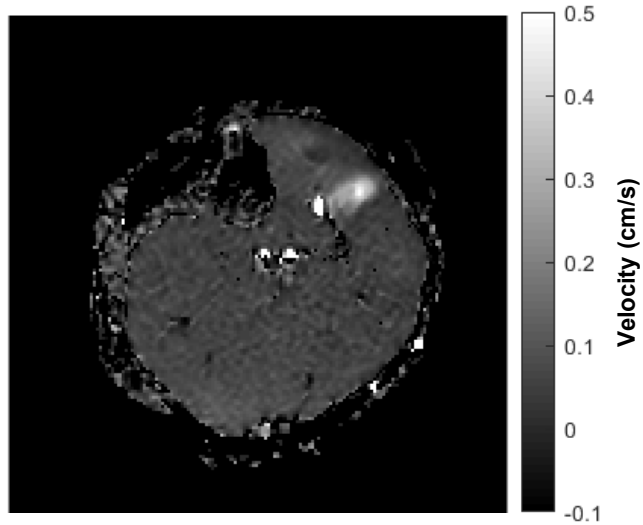**B**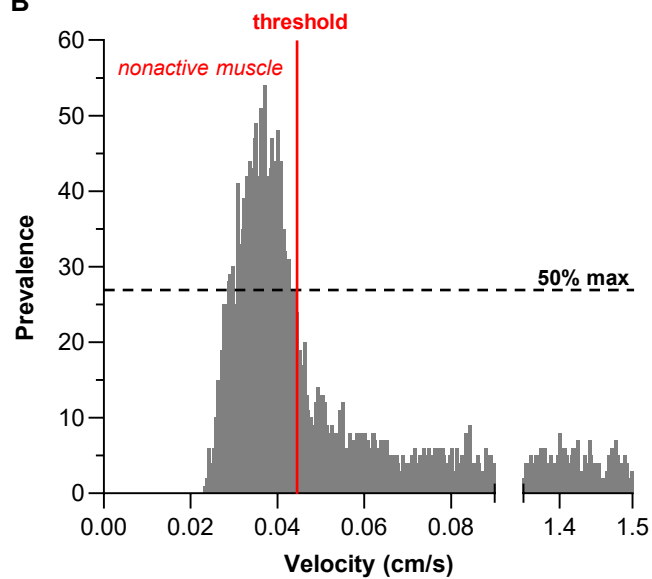**C**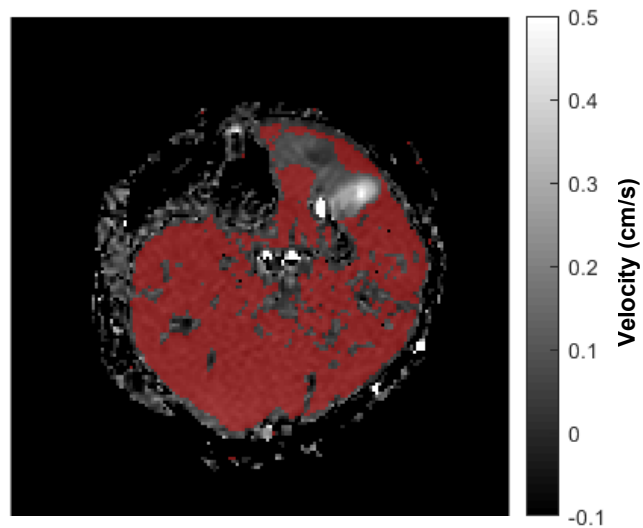**D**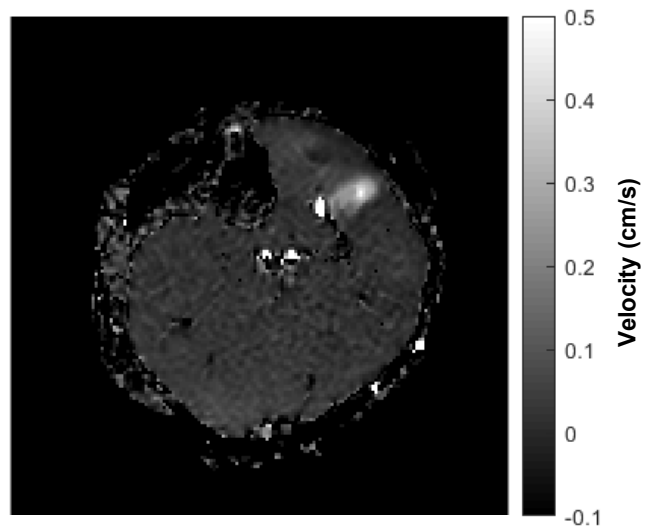

Supplement: Supplementary file 1 — Figure S1: Supporting Information [file NBM-34-e4466-s001.pdf]

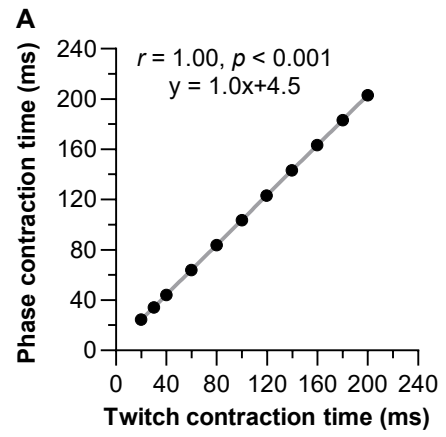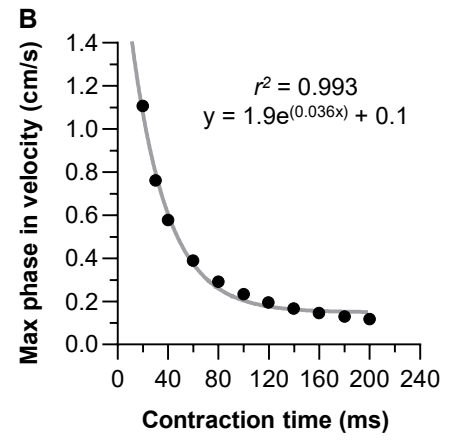

Supplement: Supplementary file 3 — Figure S3: Supporting Information [file NBM-34-e4466-s003.pdf]

# Net magnetisation

**A**

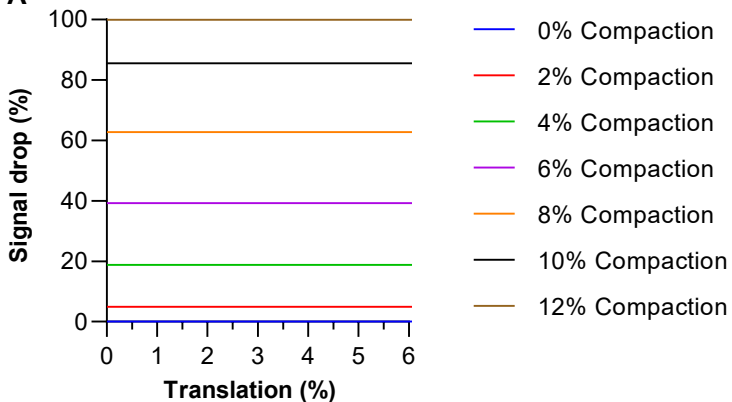

**B**

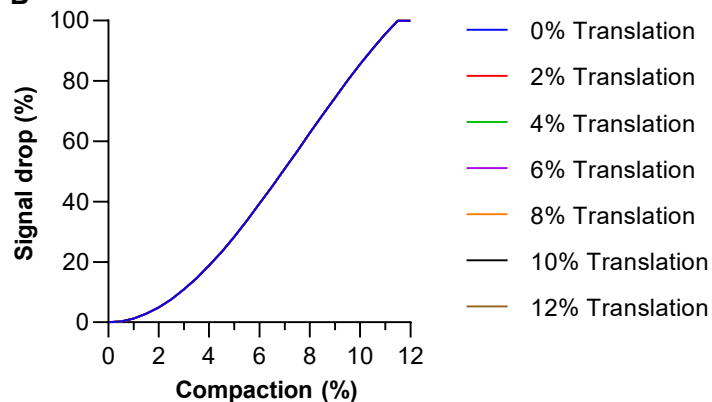

**C**

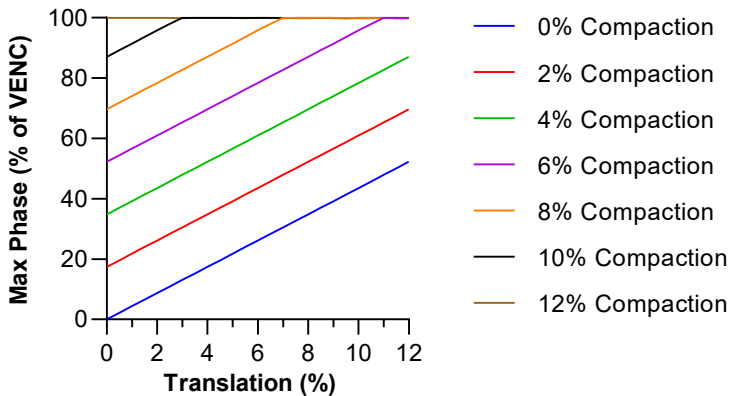

**D**

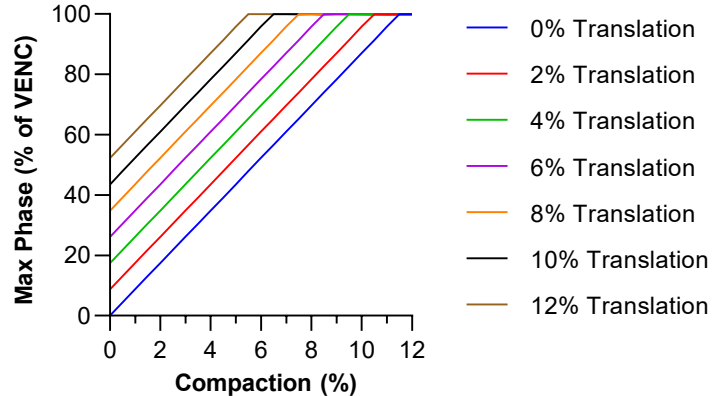

Supplement: Supplementary file 4 — Figure S4: Supporting Information [file NBM-34-e4466-s004.pdf]

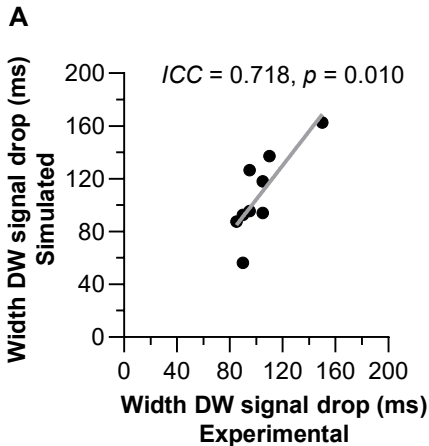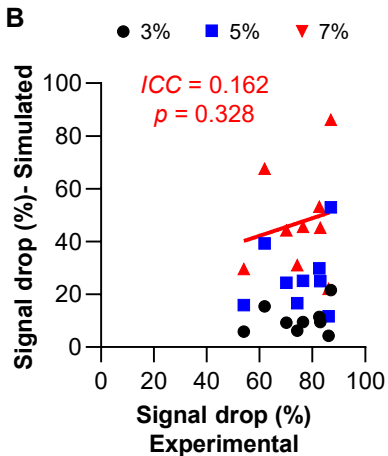

Supplement: Supplementary file 5 — Figure S5: Supporting Information [file NBM-34-e4466-s005.pdf]

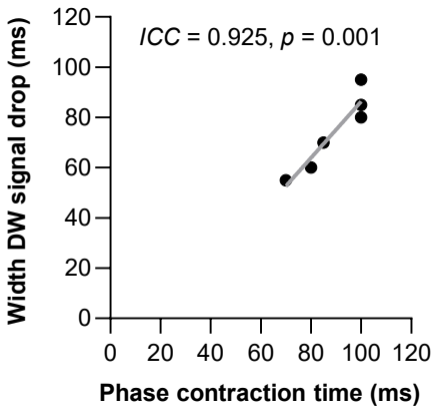

Supplement: Supplementary file 6 — Figure S6 Supporting Information [file NBM-34-e4466-s006.pdf]
