# Supplementary material for: The muscle twitch profile assessed with motor unit magnetic resonance imaging
Source: NMR Biomed. 2021 Jan 6;34(3):e4466. doi: 10.1002/nbm.4466 (PMC7900994; doi:10.1002/nbm.4466)
Supplement: Supplementary file 2 — Figure S2: Supporting Information [file NBM-34-e4466-s002.pdf]

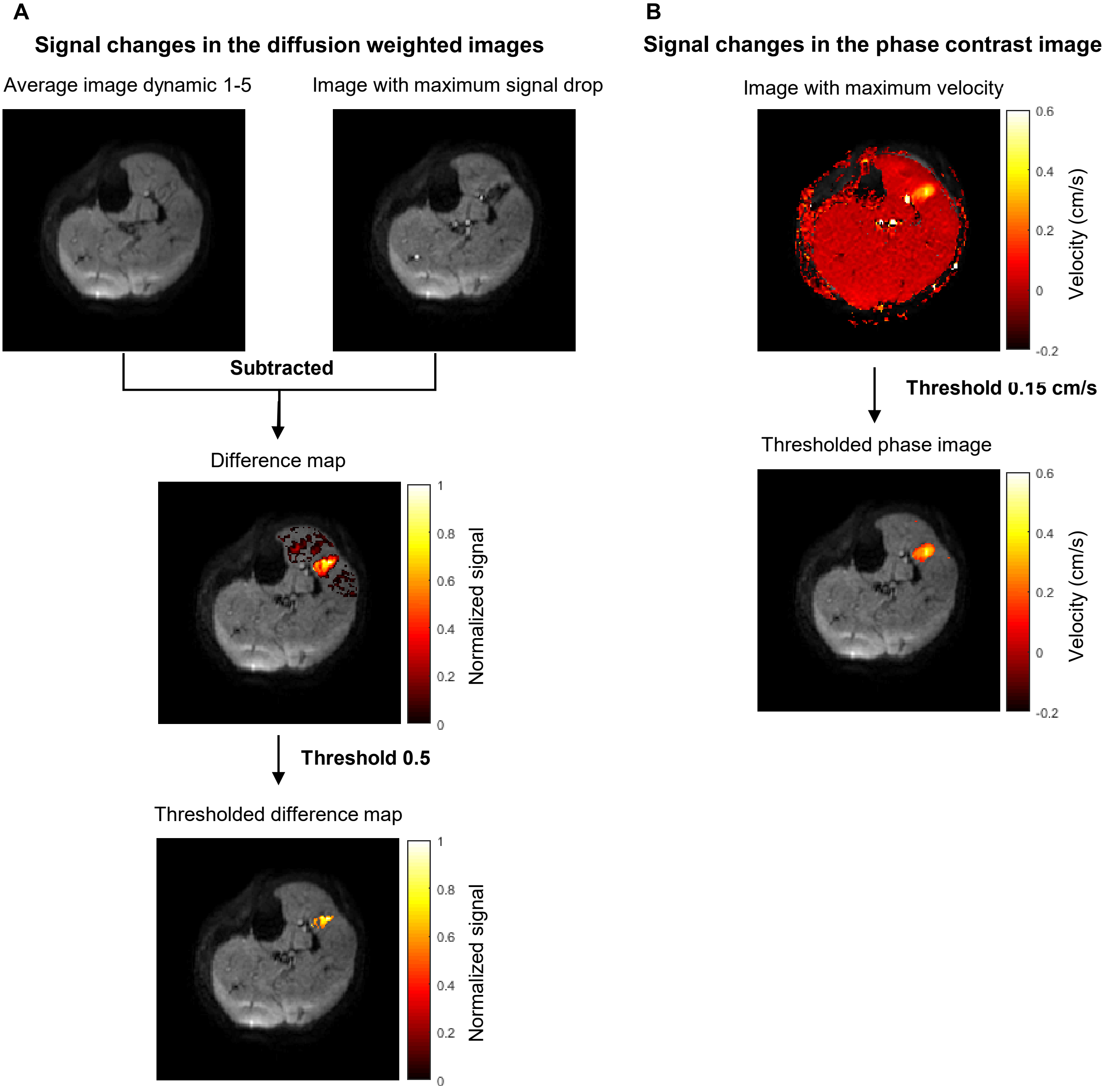
A
Signal changes in the diffusion weighted images
Average image dynamic 1-5
Image with maximum signal drop
Subtracted
Difference map
Normalized signal
1
0.8
0.6
0.4
0.2
0
Threshold 0.5
Thresholded difference map
Normalized signal
B
Signal changes in the phase contrast image
Image with maximum velocity
Velocity (cm/s)
0.6
0.4
0.2
0
-0.2
Threshold 0.15 cm/s
Thresholded phase image
Velocity (cm/s)
