# Supplementary material for: The muscle twitch profile assessed with motor unit magnetic resonance imaging
Source: NMR Biomed. 2021 Jan 6;34(3):e4466. doi: 10.1002/nbm.4466 (PMC7900994; doi:10.1002/nbm.4466)
Supplement: Supplementary file 7 — Figure S1: Phase error correction. Automatic detection of non‐active muscle. A) Original phase image whereby non‐active muscle displays a velocity off‐set (higher than 0 cm/s). B) Histogram of phase values of all voxels in the phase image of A. C) Selected non‐active muscle overlaid in red on the phase image of A. D) Baseline corrected phase image showing that non‐active muscle now has a velocity of 0 cm/s. Figure S2: Schematic overview of voxel‐by‐voxel assessment of significant diffusion weighted (DW) and phase changes for the 2D overlay. A) Overview for detection of significant DW changes. B) Overview for detection of significant phase (velocity) changes. Figure S3: Simulation results. Effect of twitch contraction time on cumulative phase signal. A) Phase contraction vs twitch contraction time. B) Maximum phase change in velocity vs. contraction time. Figure S4: Simulation results. Effect of percentage compaction and translation for asymmetric compaction. A) Percentage signal drop in net magnetisation versus percentage translation showing that percentage signal drop is independent of translation. Each line represents a percentage compaction. B) Percentage signal drop in net magnetisation versus percentage compaction. Each line represents a percentage translation showing a sigmoidal relation between percentage compaction and percentage signal drop, whereby the signal reached 100% signal drop at 11.5% compaction. C) Maximum phase expressed as percentage of VENC (1.3 cm/s) versus percentage translation showing a linear relation between percentage translation and maximum phase. D) Maximum phase expressed as percentage of VENC (1.3 cm/s) versus percentage compaction showing a linear relation between percentage compaction and maximum phase. Figure S5: Comparison of the experimental diffusion weighted (DW) signal changes and the simulated magnetisation changes using the measured force as model input. A) Width of the first DW signal drop. B) Percentage signal drop for th [file NBM-34-e4466-s007.docx]

# Supplemental materials

## Videos

The videos below are all from the same volunteer. The timing between the electrical stimulus was altered from 50 ms after the 90° radiofrequency (RF) pulse to 400 ms before the 90° RF pulse in steps of 5 ms. Every frame represents one these timing steps.

### Video 1: Latency diffusion weighted (DW) scan at stimulation current producing a visible muscle twitch (I_muscle_)

### The current used for this scan was 5.5 mA. The video shows the anterior compartment muscles go black two times in a row, the first time represents the contraction (from 0 to 3 sec) of the muscle twitch and the second time the relaxation of the muscle twitch (from 3 to 11 sec).

### Video 2: Latency diffusion weighted (DW) scan at a stimulation current activing a single motor unit (I_singleMU_)

### The current used for this scan was 4.08 mA. The video shows a single motor unit in the peroneus longus that goes black when the muscles fibres contract (from 0 to 3 sec). No second signal drop can be seen during the relaxation of the muscle fibres.

### Video 3: Latency phase contrast (PC) scan at a stimulating current activating a single motor unit (I_singleMU_)

### The current used for this scan was 4.08 mA. The video shows a single motor unit in peroneus longus. An increase in velocity (motor unit becomes white) is seen when the motor unit contracts from (0 to 3 sec) and a decrease in velocity (motor unit becomes black) when the motor unit relaxes (from 3 to 8 sec).

## Material and Methods

### Removing offset in phase contrast images

The phase contrast images contained an off-set which can be attributed to the presence of B0 eddy currents, concomitant fields and gradient non-linearity.^1^ This phase offset is depended on the velocity encoding (VENC) and removed in two steps. First, the velocity of non-active muscle was determined, non-active muscle included all voxels with a signal variance smaller than 1.5 times the most prevalent signal variance over all muscle tissue voxels (figure S1). Secondly, the velocity was further baseline corrected by subtracting the average of the first five latency steps. This velocity profile was integrated to create a displacement profile, reflecting the muscle twitch.


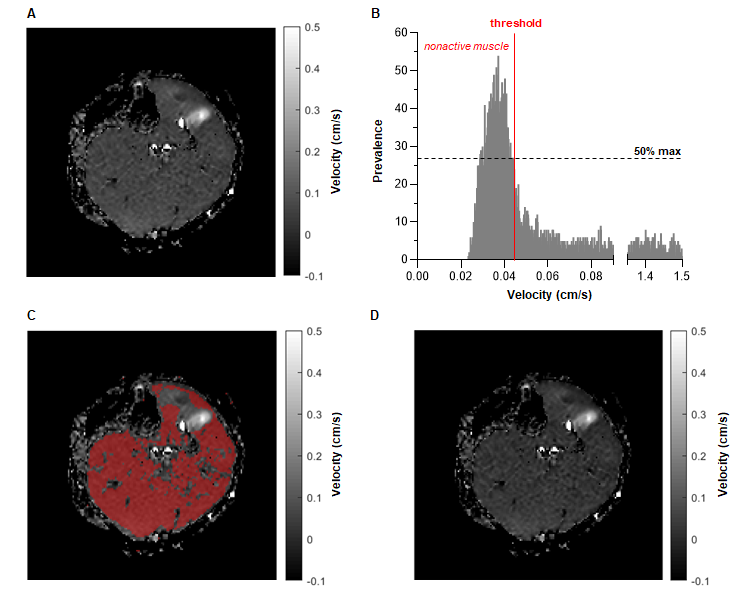


**Figure S1: Phase error correction. Automatic detection of non-active muscle.** A) Original phase image whereby non-active muscle displays a velocity off-set (higher than 0 cm/s). B) Histogram of phase values of all voxels in the phase image of A. C) Selected non-active muscle overlaid in red on the phase image of A. D) Baseline corrected phase image showing that non-active muscle now has a velocity of 0 cm/s.

### Voxel-wise analysis of diffusion weighted signal and phase contrast signal changes


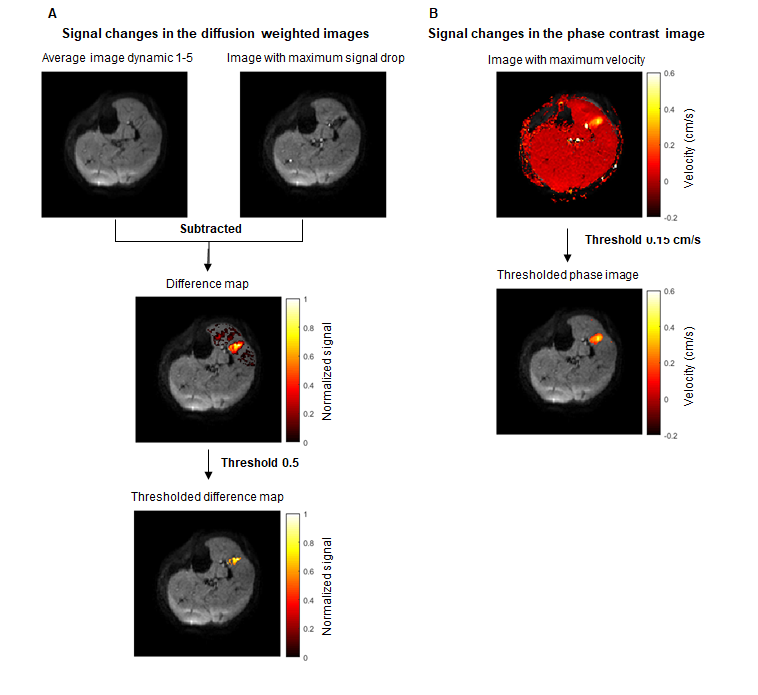


**Figure S2: Schematic overview of voxel-by-voxel assessment of significant diffusion weighted (DW) and phase changes for the 2D overlay.** A) Overview for detection of significant DW changes. B) Overview for detection of significant phase (velocity) changes.

## Results

### Simulation results: Effect of twitch contraction on cumulative phase


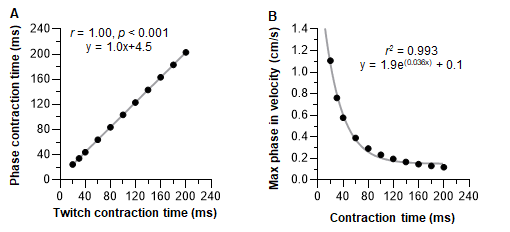


**Figure S3: Simulation results. Effect of twitch contraction time on cumulative phase signal.** A) Phase contraction vs twitch contraction time. B) Maximum phase change in velocity vs. contraction time.

### Simulation results: Effect of percentage compaction and translation for symmetric compaction


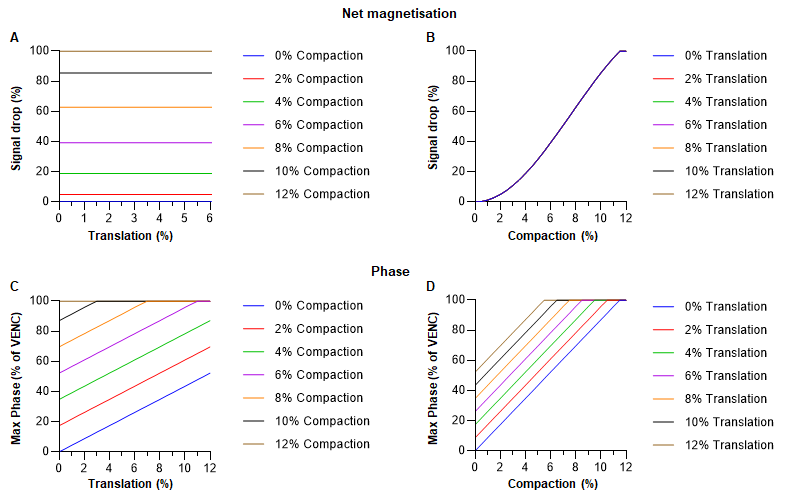


**Figure S4: Simulation results. Effect of percentage compaction and translation for asymmetric compaction.** A) Percentage signal drop in net magnetisation versus percentage translation showing that percentage signal drop is independent of translation. Each line represents a percentage compaction. B) Percentage signal drop in net magnetisation versus percentage compaction. Each line represents a percentage translation showing a sigmoidal relation between percentage compaction and percentage signal drop, whereby the signal reached 100% signal drop at 11.5% compaction. C) Maximum phase expressed as percentage of VENC (1.3 cm/s) versus percentage translation showing a linear relation between percentage translation and maximum phase. D) Maximum phase expressed as percentage of VENC (1.3 cm/s) versus percentage compaction showing a linear relation between percentage compaction and maximum phase.

### Simulated net magnetisation for experimental measured muscle twitch

**Methods:** The experimental force twitch was used as input for the theoretical model to further explore the mechanism of signal change. The simulation requires a clean noise free force signal, therefore the experimental force was modelled with a polynomial fit with 21 degrees of freedom. The net magnetisation was simulated with the PGSE-DWI gradient waveform. As the degree of muscle compaction could not be experimentally determined, the DW signal was simulated for a contraction model of 3%, 5% and 7% asymmetric compaction and no translation. The width of the simulated drop in net magnetisation and the percentage signal drop were compared with those of the measured latency DW signal.

**Results:** The width of the first drop in the DW signal of the simulated and measured latency signal were comparable (ICC=0.718, p=0.010; figure S5A). Furthermore, at least 7% compaction was needed to achieve a percentage drop in the simulated net magnetisation in same range as the percentage drop in the measured DW signal (figure 7B). However, at this 7% compaction the measured and simulated signal drop across all participants were not comparable (ICC = 0.162, p=0.328).


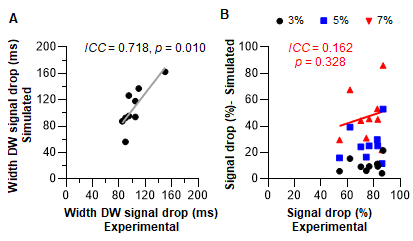


**Figure S5: Comparison of the experimental diffusion weighted (DW) signal changes and the simulated magnetisation changes using the measured force as model input.** A) Width of the first DW signal drop. B) Percentage signal drop for three levels of asymmetric compaction, 3%, 5% and 7%, and no translation, with a linear regression line for the 7% compaction.

### Phase contrast estimated contraction time vs diffusion weighted estimated contraction time


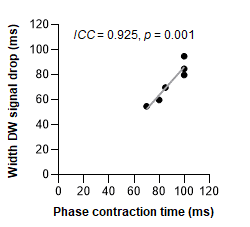


**Figure S6:** **Correlation graph of phase contrast contraction time *vs.* width of the first diffusion weighted (DW) signal drop.** Data were acquired at the stimulation current I_singleMU_ that activated single motor units.

## References

1. Gatehouse PD, Rolf MP, Graves MJ, et al. Flow measurement by cardiovascular magnetic resonance: a multi-centre multi-vendor study of background phase offset errors that can compromise the accuracy of derived regurgitant or shunt flow measurements. *J Cardiovasc Magn Reson*. 2010;12(1):5. doi:10.1186/1532-429X-12-5

2. Birkbeck MG, Heskamp L, Schofield IS, Blamire AM, Whittaker RG. Non-invasive imaging of single human motor units. *Clin Neurophysiol*. 2020;131(6):1399-1406. doi:10.1016/j.clinph.2020.02.004
